# Aggression in Women: Behavior, Brain and Hormones

**DOI:** 10.3389/fnbeh.2018.00081

**Published:** 2018-05-02

**Authors:** Thomas F. Denson, Siobhan M. O’Dean, Khandis R. Blake, Joanne R. Beames

**Affiliations:** ^1^School of Psychology, University of New South Wales, Sydney, NSW, Australia; ^2^Evolution & Ecology Research Centre, School of Biological, Earth & Environmental Science, University of New South Wales, Sydney, NSW, Australia

**Keywords:** women, aggression, brain, hormones, intimate partner violence

## Abstract

We review the literature on aggression in women with an emphasis on laboratory experimentation and hormonal and brain mechanisms. Women tend to engage in more indirect forms of aggression (e.g., spreading rumors) than other types of aggression. In laboratory studies, women are less aggressive than men, but provocation attenuates this difference. In the real world, women are just as likely to aggress against their romantic partner as men are, but men cause more serious physical and psychological harm. A very small minority of women are also sexually violent. Women are susceptible to alcohol-related aggression, but this type of aggression may be limited to women high in trait aggression. Fear of being harmed is a robust inhibitor of direct aggression in women. There are too few studies and most are underpowered to detect unique neural mechanisms associated with aggression in women. Testosterone shows the same small, positive relationship with aggression in women as in men. The role of cortisol is unclear, although some evidence suggests that women who are high in testosterone and low in cortisol show heightened aggression. Under some circumstances, oxytocin may increase aggression by enhancing reactivity to provocation and simultaneously lowering perceptions of danger that normally inhibit many women from retaliating. There is some evidence that high levels of estradiol and progesterone are associated with low levels of aggression. We highlight that more gender-specific theory-driven hypothesis testing is needed with larger samples of women and aggression paradigms relevant to women.

“…females…are not passive victims of violence. Rather, they respond to provocation and are active participants in aggressive interactions.” (Richardson, 2005, p. 245)

Aggression is a complex social behavior with many causes and manifestations. Over the past several decades, scholars have identified the many forms that aggression can take. Aggression can be physical (e.g., slapping), or verbal (e.g., shouting abuse). It can be direct in nature (e.g., directly retaliating against a co-worker) or indirect with aim of inflicting reputational harm (e.g., spreading rumors about a co-worker behind their back). Aggression can be impulsive, elicited by anger in response to provocation (known as reactive or hostile aggression) or it can be premeditated, less emotional, and used as a means to obtain some other end (known as proactive or instrumental aggression). Aggression that is physically extreme is referred to as violence (e.g., aggravated assault, homicide). Despite their apparently different surface characteristics, these instantiations of aggression all conform to the scholarly definition of aggression as behavior intended to cause harm to someone who is motivated to avoid that harm (Berkowitz, 1993; Baron and Richardson, 1994; Geen, 2001; Anderson and Bushman, 2002).

The aim of this review is to synthesize what is known about women’s aggression from behavioral and neurobiological perspectives. We first focus on the behavioral research on women-perpetrated aggression including women’s behavior in laboratory aggression paradigms, intimate partner violence (IPV), alcohol-related aggression and sexual violence. We then review data on prenatal and postnatal influences, the central nervous system, and neuroendocrine mechanisms. Figure 1 summarizes these factors. We conclude by identifying gaps in the knowledge base, and provide suggestions for future research.

**Figure 1:**
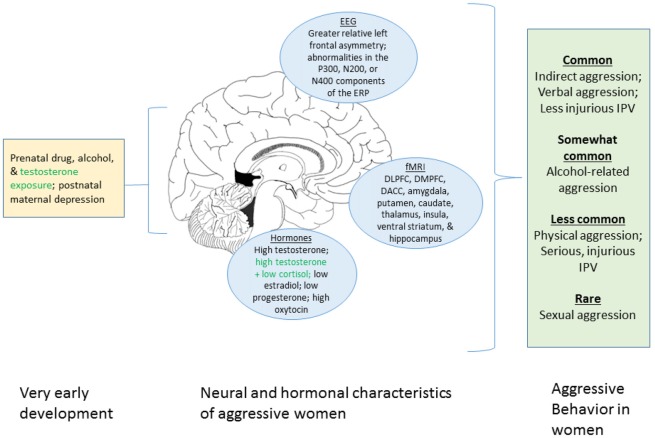
Graphical summary of the present review of factors associated with aggression in women. The left portion displays prenatal and early developmental influences known to affect aggression. The center portion shows neural and hormonal process associated with aggression in women. The right box indicates the different forms of aggression that women engage in and their relative frequencies. Green text indicates uncertainty regarding the robustness of the relationship with aggression in women. We note that this figure summarizes the current review only and that many additional factors do not appear here (e.g., genetic influences, neurotransmitter systems, societal factors). DLPFC, dorsolateral prefrontal cortex; DMPFC, dorsomedial prefrontal cortex; DACC, dorsal anterior cingulate cortex.

## Part 1: Aggressive Behavior

Compared to our knowledge of men’s aggression, relatively little is known about women’s aggression. Indeed, aggression and violence are usually considered male problems. There is some truth to this assumption. Globally, men are more violent than women (UN Office on Drugs and Crime, 2013). However, women frequently engage in other forms of aggressive behavior (Richardson, 2005). Research consistently reports that women use *indirect aggression* to an equivalent or greater extent than men (Archer and Coyne, 2005). Indirect aggression occurs when someone harms another while masking the aggressive intent (Björkqvist et al., 1992; Arnocky et al., 2012). Specific examples of indirect aggression include spreading false rumors, gossiping, excluding others from a social group, making insinuations without direct accusation, and criticizing others’ appearance or personality. Girls’ use of indirect aggression exceeds boys’ from age 11 onward (Archer, 2004). This difference persists into adulthood; compared to men, adult women use more indirect forms of aggression across various areas of life (Björkqvist et al., 1994; Österman et al., 1998). Indeed, in a large cross-cultural survey of female aggression across 317 societies, Burbank (1987) found that female aggression was mostly indirect and rarely inflicted physical injury. Thus, in the real world aggression is common in women and girls, but the form it takes is largely indirect compared to men’s aggression.

Numerous theorists have attempted to explain sex differences in aggression. Because human aggression is a complex social phenomenon elicited by multiple factors operating throughout the lifespan, one must consider how social influences interact with neurobiological mechanisms to influence aggression. Wood and Eagly’s (2002) biosocial approach suggests that sex differences in behavior (including aggression) are caused by sex differences in physical attributes that interact with cultural values and customs. They note that sex differences in physical attributes and reproduction often make it more efficient for women to perform certain tasks and for men to perform others. For instance, in their discussion of women’s historically limited involvement in warfare, they note that in most hunter-gatherer societies, men engaged in warfare more than women because men are physically larger and stronger and unable to nurse infants. Moreover, essential practices such as nursing, childcare and vegetal food production made it unlikely that women would travel far to engage in warfare (Wood and Eagly, 2002). This division of labor becomes reflected in social norms and values which are transmitted via socialization practices.

According to biosocial interactionist perspectives, social norms become relevant because most cultures endorse warfare as a means to gain status and because most cultures are patriarchal (i.e., men hold more power and status than women). Thus, most cultures reward men for being warriors and punish women for becoming aggressive. Indeed, social norms proscribe physical aggression in women (Eagly and Steffen, 1986) and girls can vocalize these norms from an early age (Crick et al., 1996). However, when women do behave aggressively and are dominant, they often face backlash against them (Barber et al., 1999). In this way, the interaction between biologically specified sex differences and sociocultural construction interact to produce lower direct aggression in women relative to men nearly everywhere in the world. In the next section, we review research on women’s laboratory aggression, alcohol-related aggression, intimate partner aggression and sexual aggression.

### Aggressive Behavior in Laboratory Studies

Psychologists have been studying aggressive behavior with laboratory aggression paradigms since the 1960s. The primary strength of laboratory aggression paradigms is that researchers can manipulate variables that might influence aggression while eliminating much of the complexity of the outside world. Researchers can then quantify the observed aggressive behavior. The most commonly used paradigms are variations of the Taylor (1967) aggression paradigm (TAP), and the point subtraction aggression paradigm (PSAP; Cherek, 1981). We first review these paradigms in order to facilitate understanding of gender differences in laboratory aggression.

#### Laboratory Aggression Paradigms

In the TAP (sometimes called the competitive reaction time task; Giancola and Zeichner, 1995b), participants are typically provoked in some manner, often through receiving electric shocks or bursts of white noise from another participant (who may be real or bogus; e.g., Giancola and Parrott, 2008; Jones and Paulhus, 2010). Participants may also be provoked by receiving negative feedback on a laboratory task such as an essay or short speech, or by being ignored, rejected, or ostracized by another person (Bushman and Baumeister, 1998; Warburton et al., 2006; Blake et al., 2018). Following provocation, participants are given the opportunity to retaliate against the provocateur to varying degrees, or respond non-aggressively. In the TAP, aggressive behavior is operationalized as the intensity and/or duration of noise blasts directed at the provocateur.

For the PSAP, participants ostensibly play a game against a real or bogus participant to earn points that may be exchanged for money. In modern versions of the paradigm, during each trial participants are given the option to either steal points, defend their points, or earn points (Geniole et al., 2017). Provocation is induced when the focal participant has points stolen from them by the other participant, and aggression is observed when the focal participant steals money from the other participant. As in the TAP, participants may also be provoked via insulting feedback or ostracism. In addition to the TAP and PSAP, aggression in the laboratory can also be operationalized by giving the experimenter a poor recommendation for a coveted job (e.g., Denson et al., 2011) and giving hot sauce to a participant who is known to dislike spicy foods (Lieberman et al., 1999). However, the TAP and PSAP are the most widely studied.

Some researchers have criticized laboratory aggression paradigms on the grounds of poor external validity (e.g., Tedeschi and Quigley, 1996; Ritter and Eslea, 2005). It is true that laboratory paradigms lack a superficial similarity to the real world (i.e., mundane realism). However, several researchers have quantitatively shown that laboratory paradigms possess both strong psychological realism and external validity (Anderson and Bushman, 1997; Giancola and Chermack, 1998; Giancola and Parrott, 2008). For instance, female parolees with a violent criminal history steal more points in the PSAP than non-violent parolees (Cherek et al., 2000). Importantly, all laboratory aggression paradigms are consistent with the widely accepted definition of aggression as behavior intended to harm another person (Anderson and Bushman, 2002). However, few studies were specifically designed to externally validate laboratory aggression paradigms with women.

#### Meta-Analytic Evidence

To date, there have been three large scale meta-analyses of gender differences in laboratory aggression paradigms (Eagly and Steffen, 1986; Bettencourt and Miller, 1996; Bettencourt and Kernahan, 1997)^1^. Consistent with the social psychological *Zeitgeist* at the time, Eagly and Steffen (1986) favored a social learning explanation of gender differences over biological explanations. They concluded that women are less aggressive than men because social roles encourage aggression in men but not women. They found a small-to-medium effect such that men were more physically aggressive than women (*d* = 0.40), but this effect was greatly reduced for non-physical forms of aggression such as verbal aggression (*d* = 0.18). A separate group of 200 men and women coded how they would feel if they were to aggress in each study included in the meta-analysis. Relative to men coders, women coders anticipated that experiencing greater guilt, anxiety, and danger would be the consequences of aggressing. Thus, women may be less likely to aggress in the laboratory due to fear of retaliation and an unwillingness to harm others.

In what still remains the most comprehensive meta-analysis to date of gender differences in laboratory aggression, Bettencourt and Miller (1996) examined 107 effect sizes from 64 experiments. Overall, they found a small gender effect (*d* = 0.24) such that men were somewhat more aggressive than women. When unprovoked, women were less physically and verbally aggressive than men. However, provocation attenuated the gender difference in physical aggression and ameliorated the gender difference in verbal aggression.

Bettencourt and Miller (1996) also examined whether the type of provocation would influence gender differences in aggression. They found that men were more aggressive than women when the provocation induced frustration or insulted participants’ intelligence. By contrast, the gender difference in aggression was reduced to zero in studies that manipulated provocation with physical attack (e.g., electric shocks) or an insulting evaluation (e.g., on an essay task). Thus, women and men may be equally aggressive when faced with physical attack or an unjustified insult, at least in the laboratory. Consistent with Eagly and Steffen (1986), Bettencourt and Miller (1996) found that women coders anticipated greater danger than men coders were they to aggress and that men perceived the provocation as more intense than women. These perceptions subsequently predicted a greater male-biased gender difference in aggression. Thus, both meta-analyses converged on perceived danger as one putative psychological gender difference that explains lower aggression observed in women in the laboratory.

During the “cognitive revolution” in social psychology in the 1970s and 80s, many researchers were influenced by Berkowitz’s (1993) and Berkowitz and LePage’s (1967) cognitive neoassociationistic theory of aggression. According to the theory, any aggression-related cues (e.g., weapons, violent images, hostile words, alcohol) prime a cognitive network of aggression-related associations. These primed associations increase aggression when provoked (Carlson et al., 1990). A meta-analysis of the violent cue literature found that men were moderately (*d* = 0.41) more aggressive than women when participants were not provoked but exposed to violent cues (Bettencourt and Kernahan, 1997). The putative cause is that men may have a more extensively developed violence-related cognitive network than women, possibly due to gender norms that encourage male aggressiveness (i.e., social role theory). Women may behave less aggressively because female gender roles stipulate that women should not act aggressively when unprovoked. However, as in the previous meta-analyses, this gender difference was reduced to zero when participants were provoked. The authors concluded that once provoked, the influence of gender roles on aggression may become less salient.

#### Alcohol-Related Aggression

Alcohol-related aggression is of interest to neuroscientists because acute and chronic alcohol use is thought to increase risk for aggression via dysfunction in the prefrontal cortex (PFC; Giancola, 2000; Heinz et al., 2011; Gan et al., 2015). Alcohol is also involved in a large proportion of violent crimes. For instance, a study of 11,836 men and women arrested for violent crimes found that 57% and 44% had been drinking prior to committing the crime, respectively (Martin and Bryant, 2001). Similarly, relatively greater alcohol consumption equally predicted fighting in a young group of British men and women (Wells et al., 2005). Although the effects of alcohol on facilitating aggression in men has been an active research area, the same cannot be said for research with women.

In terms of laboratory research, two meta-analyses examined the literature on alcohol-induced aggression that manipulated alcohol administration (Ito et al., 1996; Bushman, 1997). The mean effects of alcohol on aggression were significant for men but not for women; however, both meta-analyses were underpowered to detect effects in women. Nonetheless, several individual experiments have examined alcohol-induced aggression in women.

In two separate experiments, men and women were assigned to a low dose or high dose of alcohol or tonic (Rohsenow and Bachorowski, 1984). To control for expectancies about the effects of alcohol on aggression, half of the participants were told they received alcohol and the other half tonic. They were then insulted by another fictitious participant and given the opportunity to retaliate via a harsh written evaluation (i.e., a measure of verbal aggression). For women, alcohol increased aggression, but only at the low dose. In another experiment, participants consumed alcohol on one day and placebo on another day (Dougherty et al., 1999). On both days, they played the PSAP against a fictitious participant six times from morning to afternoon. Relative to the placebo day, alcohol increased aggression in both men and women for several hours. Furthermore, men and women who were more aggressive on the placebo day showed the greatest alcohol-related aggression. This latter finding suggests that people with dispositions toward aggression when sober are more likely to become aggressive when intoxicated.

Several experiments support this notion that dispositional aggression is related to alcohol-induced aggression. For instance, Giancola (2002a) found that men and women high in dispositional aggressiveness exhibited alcohol-induced aggression in the TAP, but the relationship between trait aggression and aggressive behavior was stronger for men than women. Another analysis of the same sample found that dispositional anger was also positively correlated with alcohol-induced aggression in women, but only at low provocation levels (Giancola, 2002b). For men, trait anger was positively correlated with aggressive behavior and with low and high levels of provocation. Despite a general tendency for alcohol intoxication to increase aggression in men and women, several other studies found that alcohol did not increase aggression in women, but did in men (e.g., Giancola and Zeichner, 1995a; Hoaken and Pihl, 2000; Hoaken et al., 2003; Gussler-Burkhardt and Giancola, 2005).

A recent meta-analysis was able to provide somewhat stronger evidence for the aggression-augmenting effect of alcohol in women (Crane et al., 2017). They examined the 12 available alcohol administration experiments that included women and found a small, but significant effect of acute alcohol intoxication on increased aggression, *d* = 0.17, CI_95_ = 0.03, 0.30. This effect is smaller than that observed in men (i.e., *d*s = 0.49 and 0.50, in Ito et al. (1996) and Bushman (1997)). However, with only 12 experimental studies on alcohol-related aggression in women, more research is needed both in the laboratory and natural settings.

A follow-up meta-analysis examined gender of the target and provocation as moderators of alcohol-induced aggression in women (Crane et al., 2018). Alcohol increased aggression in women in studies that used relatively more intense provocation (e.g., shocks, insults) but not in studies that used more innocuous provocations (e.g., reading upsetting vignettes). Alcohol increased aggression in studies that included female targets, but not male targets. Although the number of studies was small in number (*k* = 14), these findings suggest that alcohol-related aggression in women may be strongest when provoked and retaliating against women targets.

#### Summary of the Laboratory Research

The extensive experimental literature on aggression in women and men provides a solid evidence base for the primary conclusion that women are less physically aggressive than men. This finding is consistent with crime statistics showing that men are by far the most violent gender. Nonetheless, women are capable of behaving aggressively, especially when provoked. The gender difference in aggression becomes much smaller when participants are provoked in the laboratory and non-existent when participants are allowed to verbally aggress (Bettencourt and Miller, 1996). Women’s relatively lower aggression when unprovoked seems at least partially attributable to greater fearfulness than men when considering behaving aggressively (Eagly and Steffen, 1986; Bettencourt and Miller, 1996). For instance, in one experiment exposure to a laboratory stressor increased aggression in men but decreased aggression in women (Verona and Kilmer, 2007). The authors suggested that women may experience a withdrawal reaction in stressful circumstances whereas men are more likely to experience an approach response.

The research on alcohol-related aggression suggests that intoxication increases aggression in men and women, but the effect tends to be larger in men and people with pre-existing dispositions toward aggressive behavior. Alcohol-related aggression in women tends to be most severe when provoked and the target of aggression is a woman. One limitation of the laboratory research is that conclusions are based largely on just two direct aggression paradigms: the TAP and PSAP. Although these are well-validated paradigms, the field could benefit from a more diverse set of paradigms. For instance, experimental alcohol research with women and indirect aggression would be informative.

### Intimate Partner Violence

Conflict, especially around romantic jealousy, can elicit aggression between partners, which is known as IPV. Prevalence and victimization rates vary substantially depending on the methodology used and population sampled. Definitions also vary, but in the IPV literature, IPV is frequently considered to be any act of aggression directed toward one’s partner, rather than violence specifically (i.e., extreme acts of physical aggression). Lifetime prevalence of IPV victimization was estimated at 37.3% for women and 30.9% for men living in the United States (Smith et al., 2017). Between 8% and 21% of a representative sample of American couples reported experiencing at least one act of IPV in the past year (Schafer et al., 1998). In this section, we focus on heterosexual relationships as relatively little is known about IPV in same-sex attracted relationships in women (for an exception, see Badenes-Ribera et al., 2016).

Some women do use violence against their romantic partners, although the severity and form of the IPV may differ compared to male-perpetrated IPV. Women tend to engage in fewer acts of severe IPV than men, just as women engage in less aggression than men generally. For instance, one study of IPV arrestees reported that women used an average of 1.44 severely violent tactics (as defined by the severe violence scale of the Conflict Tactics Scale; Straus, 1979) during the arrest incident, whereas men used an average of 2.27 severely violent tactics (Busch and Rosenberg, 2004). Women are more likely than men to throw objects at their victim, to use weapons, and to bite their victims (Magdol et al., 1997; Archer, 2002; Melton and Belknap, 2003), whereas men are more likely to beat up, choke or strangle their victims (Archer, 2002).

These gender differences in IPV-related violence are likely due to sexual dimorphism in physical attributes. Because of men’s greater size and strength relative to women, on average women can inflict more harm with weapons and thrown objects than their bodies, whereas men can inflict equivalent or greater harm with their bodies. Indeed, IPV causes visibly greater physical and psychological harm in women than men (e.g., Morse, 1995; Archer, 2000; Caldwell et al., 2012). A meta-analysis found that male IPV perpetrators were more likely to cause physical injury than female IPV perpetrators. Over 60% of those injured by their partners in an IPV incident were women (Archer, 2000). Female victims of IPV are not just more likely to suffer physical injury, but also posttraumatic stress disorder, depression and anxiety than their male counterparts (Caldwell et al., 2012). Additionally, an important aspect of IPV is sexual violence, and in this category, women are far more likely to be victims than men (Foshee, 1996; Coker et al., 2002; Black et al., 2011). Similarly, women are far more likely to be victims of IPV-related homicide than men. In Australia, women comprise 76% of IPV homicide victims (Ramsey, 2015). Other countries also show this gender disparity in IPV homicide rates. The World Health Organization (2013) examined 1121 crime data estimates of IPV-related homicides across 65 countries from 1982 to 2011. Of these homicides, the median prevalence of women killed by their partners was 38%, whereas the corresponding rate of murdered men was 6%.

Not all research found lower use of severe violence in women. Some studies using data from the criminal justice system (e.g., police reports, pretrial information and victim statements) of IPV offenders highlight commonalities regarding the use of IPV in women and men. These studies reported that defendants of both genders are equally likely to engage in harassing behavior (e.g., trespassing and stalking), and to have been physically abusive by punching, hitting, slapping, or stabbing (Melton and Belknap, 2003). Findings from these forensic studies suggest women are equally likely to use severe forms of violence as men and to severely injure their partners (e.g., Melton and Belknap, 2003; Busch and Rosenberg, 2004; Henning and Feder, 2004). Several other studies reported that both men and women use coercive and controlling behavior against their partners in equivalent rates (Stets and Pirog-Good, 1990; Stets, 1991; Felson and Outlaw, 2007; Hines et al., 2007; Straus and Gozjolko, 2014), but other studies found that women are less likely than men to engage in controlling behavior (e.g., Johnson, 2006; Hester, 2009).

The criminology literature also highlights important gender differences. For instance, women are more likely than men to be involved in “dual-arrests” (i.e., both partners are arrested at the same time; Melton and Belknap, 2003; Henning and Feder, 2004). The authors concluded that dual-arrests might provide evidence for the proposition that many women who commit IPV do so in self-defense (Melton and Belknap, 2003). Women are also much less likely than men to have repeat offenses documented (Hester, 2009), and less likely to have violated an existing protection order (Henning and Feder, 2004). Additionally, based on 16 empirically validated risk factors for criminal recidivism, male IPV perpetrators presented a greater concern for future violence than female perpetrators. Specifically, female perpetrators ranked higher on only three risk factors; younger age, unemployment and severity of offense (i.e., more likely to have used a weapon). By contrast, male offenders ranked higher on the remaining 13 risk factors, including escalation of conflict frequency and/or severity, threats to kill and substance abuse (Henning and Feder, 2004).

Data from the criminal justice system may not generalize to the wider population. IPV offenders who have become involved with law enforcement may differ in numerous ways from those who have not become involved in the criminal justice system. Indeed, large scale studies find that women and men perpetrate IPV (i.e., any form of aggression directed at their partner) at similar rates, although the severity and types of aggressive acts may differ (e.g., Straus, 1980; Archer, 2000; Gass et al., 2011; Desmarais et al., 2012; Renner and Whitney, 2012; Hamel et al., 2015).

These similar rates of IPV perpetration are likely due to the bidirectional aggression that occurs during episodes of IPV. Bidirectional IPV occurs when each partner is both a perpetrator and a victim of IPV (Mennicke and Wilke, 2015). A review of 50 studies examining self-report, police report and archival data studies found that between 49.2% and 69.7% of IPV was bidirectional (Langhinrichsen-Rohling et al., 2012). This bidirectional nature of IPV is also consistent across diverse populations. A review of 111 articles found that female IPV perpetration tends to be highest in clinical populations, with a 41.7% pooled prevalence rate, and lowest in large population studies with a 24.1% pooled prevalence rate (Desmarais et al., 2012), Although the average prevalence rates of women perpetrating IPV differ significantly across different populations (for review see Desmarais et al., 2012; Langhinrichsen-Rohling et al., 2012), the proportion of bidirectional IPV remains consistent across diverse samples, averaging 57.5% (Langhinrichsen-Rohling et al., 2012).

#### Motivations for IPV Perpetration

Studies on female perpetrators of IPV show that risk factors and motivations for violence are heterogeneous. One systematic review article focused on motivations for women’s IPV perpetration (Bair-Merritt et al., 2010). The review of 23 studies found that self-defense, expressing anger, control, desire for the partner’s attention, and retaliation motivated women’s IPV perpetration. Indeed, being victimized by an intimate partner is consistently one of the strongest predictors of IPV perpetration for both men and women (O’Leary and Slep, 2012).

One study examined whether motivations for IPV perpetration in women could predict more or less engagement in IPV (Caldwell et al., 2009). Motivations included negative emotion expression, control, jealousy and wanting to portray “toughness” to ward off potential victimization. Each motivation significantly predicted physical aggression towards male partners, even when controlling for prior victimization. Likewise, control, toughness portrayals, and negative emotion expression were predictive of psychological aggression perpetration. Jealousy and control motives were also positively predictive of coercive, controlling IPV perpetration (Caldwell et al., 2009).

A more recent study examined the motives for IPV in both men and women arrested for domestic violence offenses (Elmquist et al., 2014). This study found that men and women perpetrators were equally motivated by self-defense, communication difficulties, power/control, and jealousy. Women were, however, significantly more likely to cite negative emotion expression and retaliation as reasons for engaging in IPV than men (Elmquist et al., 2014).

Furthermore, a recent meta-analysis of 580 studies identified 60 risk-factors for IPV perpetration across four different categories; demographic markers, family-of-origin markers, relationship markers and mental health/individual markers (Spencer et al., 2016). Of these 60 risk factors, only three differed by gender. Specifically, alcohol use/abuse was a significantly stronger risk-factor for male than female IPV perpetration. Secondly, a demand/withdrawal relationship communication style was a significantly stronger risk factor for IPV perpetration for men than for women. Finally, experiencing or witnessing domestic abuse as a child was a stronger risk factor for men than for women (Spencer et al., 2016). In sum, the existing literature illustrates more similarities than differences in the motivations and risk factors for IPV perpetration of men and women. These motivations and risk factors could be considered in the development of IPV prevention programs for both men and women.

#### Treatment for Women IPV Perpetrators

One out of 10 clients in batterer intervention programs are women (Price and Rosenbaum, 2009), and women often find themselves in batterer intervention programs that were designed for men (Goldenson et al., 2009). These existing programs (e.g., Duluth group therapy and cognitive behavioral therapy) have little to no effect on IPV recidivism in male offenders (Babcock et al., 2004; Stover et al., 2009). Few studies have examined the effectiveness of batterer intervention programs on female perpetrators (Carney et al., 2007). One such study found women were less likely to be physically abusive and passive-aggressive to their partners at the end of treatment; however, their likelihood of using controlling behavior remained unchanged (Carney and Buttell, 2004). Growing evidence for equivalent rates of IPV perpetration among women and men and the lack of studies on batterer intervention programs with women highlights the need for research on interventions for all IPV offenders.

#### Summary

There is ample evidence to suggest that women are as likely, if not more likely than men, to commit IPV (e.g., Archer, 2000). However, research also suggests that male perpetrated IPV is more likely to cause physical and psychological injury to women (e.g., Archer, 2000; Caldwell et al., 2012). Studies have also found that male perpetrators of IPV commit a higher number of severely violent acts (Busch and Rosenberg, 2004), and historically have more IPV offences documented than women perpetrators (Hester, 2009). The existing literature emphasizes that IPV is a complex phenomenon that arises from multiple risk and motivational factors (e.g., Elmquist et al., 2014; Spencer et al., 2016). There is little evidence that these factors differ between genders. Thus, it is critical that future research tests what are perhaps simplistic assumptions about male and female IPV perpetration (Richardson, 2005). Namely, the assumptions that men perpetrate IPV to control women, and that women perpetrate IPV only in self-defense (Spencer et al., 2016). Despite potential differences in IPV perpetration by men and women, it is important to also consider women’s role in aggressive relationships. Without doing so, there is less room for the development of effective prevention strategies for couples experiencing IPV.

### Sexual Aggression

Like most other forms of aggression, men are more likely to perpetrate sexual aggression than women. One in six American women is raped during their lifetime, the vast majority by men (Centers for Disease Control, 2010). In Australia, 19% of women have experienced sexual violence since age 15 (Parliament of Australia, 2006). Nonetheless, a small minority of women commit acts of sexual aggression against men, women and children. Sexual aggression encompasses numerous sexual activities forced upon a victim without the victim’s consent (Krahé and Berger, 2013). As is the case with men, acts of sexual aggression committed by women may include coerced sex, anal or vaginal penetration, oral sex, kissing, exposing genitals and using objects to cause harm (Krahé and Berger, 2013; Cortoni et al., 2017).

A recent meta-analysis examined the prevalence rate of female sexual offending from 2000 to 2013 in 12 countries (Australia, Belgium, Canada, England and Wales, France, Ireland, New Zealand, Norway, Scotland, Spain, Switzerland and the United States; Cortoni et al., 2017). Rather than relying on selected samples, the authors included official government crime statistics and large scale surveys that examined victimization. Results showed that 2.2% of sexual offenders were women. Girls were more likely to perpetrate than adult women. Approximately 40% of victims were men and 4% women. In two-thirds of cases, women were the sole perpetrators. The remaining offenders co-perpetrated, mostly with a man (Budd et al., 2017).

As with male-perpetrated sexual aggression, female-perpetrated sexual aggression is likely to go unreported to police (Stemple et al., 2017). For instance, the Cortoni et al. (2017) meta-analysis reported the prevalence of victimization at approximately 11%, which was 5–6 times higher than the offender prevalence rate derived from the crime statistics. This discrepancy suggests that victims of female-perpetrated sexual aggression are unlikely to report the crime to police. Victims may fear blame, social sanctions, humiliation, or that their accusations may not be taken seriously by professionals (Fisher and Pina, 2013; Stemple et al., 2017).

Because alcohol is involved in most instances of sexual aggression, victims may blame themselves for drinking. In one large scale survey of German university students, nearly 70% of women perpetrators reported that one or both partners drank alcohol prior to offending (Krahé and Berger, 2013). As do men, women perpetrators report encouraging their victims to use alcohol and take advantage of their victim’s intoxicated state (Struckman-Johnson et al., 2003). Thus, alcohol plays a substantial role in women’s sex offending and probably underreporting as well.

#### Summary

Sexual aggression is primarily perpetrated by males. Nonetheless, a small group of women are sexually aggressive. There is relatively little understanding of why some women perpetrate sexual aggression. Theoretical development should be a priority for this area. Feminist theories of male-perpetrated sexual aggression suggest that men commit rape out of patriarchal concerns and to control women (Brownmiller, 1975). Application of these theories to women may not be appropriate. As the data to date are descriptive and correlational, experimental research with laboratory sexual aggression paradigms is needed to identify causal influences and moderators. Such paradigms exist but to our knowledge have not yet been used with women (for a review, see Davis et al., 2014).

## Part 2: Neurobiological Pathways to Women’s Aggression

In this section, we review data on prenatal and postnatal influences, the central nervous system, and neuroendocrine mechanisms that may affect women’s aggression.

### Prenatal and Postnatal Influences

Gender differences in aggression emerge during toddlerhood (Archer, 2004). Thus, one approach to understanding these differences is to examine the earliest possible developmental time periods: the prenatal and postnatal periods. The idea is that exposure to certain social or biological risk factors during these sensitive developmental periods can disrupt the normal development of the nervous system, which may predispose offspring to aggression later in life. Here we selectively review a subset of some of the more widely studied factors that have been examined within the context of female aggression in humans and rodents.

Despite showing that exposure to several factors increases risk for aggression during development, there has been limited success in identifying distinct neurobiological pathways to aggression for girls and boys. Liu (2011) reviewed a number of prenatal, perinatal, and postnatal risk factors including smoking during pregnancy, birth complications, maternal depression, malnutrition, lead exposure, head injury, child abuse and maternal stress. Of these, there was only evidence for gender differences in two risk factors: maternal malnourishment and maternal depression. Sons, but not daughters of malnourished mothers were 2.5 times more likely to be classified with antisocial personality disorder as adults. There were also gender differences in the effects of maternal depression on externalizing behavior (i.e., disruptive behavior which includes aggression). For instance, a longitudinal study of over 1,300 children and their mothers found that greater maternal depression was associated with greater externalizing behavior in boys at 2 years of age, but the relationship was stronger for girls at 6 years of age than boys (Blatt-Eisengart et al., 2009). Although the reason is unclear, the disruption to caregiving caused by maternal depression may be particularly difficult for older daughters.

Research with rodents and humans has shown effects of prenatal exposure to psychotropic substances such as cannabis, nicotine, cocaine, and alcohol on aggression in female offspring. For instance, one study found that prenatal cannabis exposure was associated with aggression in 18-month old girls (El Marroun et al., 2011). Another study found that prenatal smoking positively predicted aggression in girls aged 17–42 months, although girls remained less aggressive than boys (Huijbregts et al., 2008). Prenatal cocaine exposure in 5-year olds also increased aggression, but less so in girls than boys (Bendersky et al., 2005). Another study found that prenatal exposure to cocaine predicted heightened aggression in 6–7 year old girls but not boys, and only among girls who had not been exposed to alcohol prenatally (Sood et al., 2005). The rodent literature does not suggest robust gender differences. The increased aggression induced by prenatal cocaine exposure persists into adulthood in both male and female rodents (Williams et al., 2011).

Consistent with these findings, in one study participants played a modified version of the PSAP in which they could not only aggress or earn points, but also temporarily escape. This study included a group of teens with little or no prenatal cocaine exposure and another group of teens with heavy prenatal exposure (Greenwald et al., 2011). The groups did not differ in aggression but the heavy exposure group was more likely to use the escape option. Girls were even more likely than boys to choose the escape option. Thus, prenatal cocaine exposure may alter both flight and fight responses in girls later in life.

As with alcohol use in adulthood, prenatal alcohol exposure has a large body of evidence supporting its role in increasing aggression later in life. For instance in one large-scale study of 625 families, 6–7 year old children who had been exposed to prenatal alcohol were more aggressive (Sood et al., 2001). Girls were less aggressive than boys. The rodent literature suggests that prenatal alcohol exposure increases aggression in male rats but can increase or decrease aggression in female rats (Marquardt and Brigman, 2016).

Prenatal testosterone exposure may also be a developmental mechanism underlying aggression in women. For instance, congenital adrenal hyperplasia is characterized by overproduction of androgens including testosterone in the prenatal environment. Girls and women with this condition are more physically aggressive than girls and women without this condition (Hines, 2010). Studies with rodents also typically show that prenatal testosterone exposure increases aggression in both males and females (e.g., vom Saal, 1979; Mann and Svare, 1983). Other human work has tested the twin testosterone transfer hypothesis, which is the notion that same-sex girl twins should have lower levels of testosterone exposure prenatally than opposite-sex twin pairs (Tapp et al., 2011). This increased testosterone is thought to heighten aggressiveness in the girls who shared the prenatal environment with their brother. One study of 13 year-old twins found support for this notion (Cohen-Bendahan et al., 2005), but robust evidence for this hypothesis is lacking (for a review, see Tapp et al., 2011). Similarly, the ratio of the second finger length to fourth finger length (i.e., 2D:4D ratio) is considered an indirect indicator of prenatal testosterone exposure. Smaller values are thought to indicate higher prenatal testosterone exposure. A meta-analysis showed no relationship between the 2D:4D ratio and aggression in women and only a small but significant effect in men for verbal aggression only (*r* = 0.035; Turanovic et al., 2017). Thus, the evidence for prenatal testosterone as a risk factor for women’s aggression is mixed.

#### Summary

Several prenatal and postnatal influences heighten risk for aggression later in life, but most do not differentiate between males and females. Of the risk factors reviewed here, the most evidence for sex-dependent effects is for postnatal maternal depression, prenatal maternal malnourishment, and prenatal exposure to drugs and alcohol. There is some evidence for prenatal testosterone exposure increasing aggression in girls later in life, but the evidence is mixed.

### Brain

In recent decades, researchers have made use of electroencephalography (EEG), brain stimulation, physical body manipulations and functional magnetic resonance imaging (fMRI) to examine the neural mechanisms underlying aggression. We review some of the evidence that examined both women and men or women only. We mention gender differences only when they were reported in the source articles.

#### EEG

##### State and Trait Anger/Aggression Correlate With Resting Frontal Asymmetry

Relatively greater left resting frontal alpha asymmetry is an indicator of approach motivation and greater right asymmetry is an indicator of avoidance motivation (Harmon-Jones et al., 2010). Anger and aggression are considered approach-related phenomenon (Carver and Harmon-Jones, 2009). Several studies indicate that greater individual differences in resting left frontal alpha asymmetry are positively correlated with dispositional anger (e.g., Harmon-Jones and Allen, 1998; Harmon-Jones, 2004; Hewig et al., 2004). Left-sided frontal asymmetry is also positively correlated with trait aggression. For example, in a sample of 15 boys and 11 girls, Harmon-Jones and Allen (1998) found a small, although non-significant, positive correlation between relative left frontal activation and trait aggression. Thus, individual differences in anger and aggression are linked to this neurophysiological indicator of approach motivation.

In a study of 30 men and 35 women, the authors examined the extent to which trait anger and two types of dispositional anger expression styles correlated with resting frontal asymmetry (Stewart et al., 2008). The anger expression styles referred to the extent to which people tend to express anger and aggression (i.e., anger-out) or suppress anger and aggression (i.e., anger-in). Higher trait anger was associated with greater relative left mid-frontal asymmetry. For participants high in trait anger, anger-in (rather than anger-out) positively correlated with relative left activation in regions other than the frontal cortex. Results remained significant even when gender was included as a covariate, suggesting that differences between men and women did not overly influence the correlations in this study. Experimental studies that manipulated state anger conceptually replicated and extended the initial correlational work on trait anger and relative left frontal asymmetry (for a review see Harmon-Jones and Gable, 2017).

##### Trait Anger/Aggression Correlate With Event-Related Potentials (ERPs)

Additional research investigated the relationship between trait anger or aggression and electrical activity in response to stimuli (for an overview, see Flannery et al., 2007). This electrical activity is known as an event-related potential (ERP). ERP studies of aggression have primarily used oddball or continuous performance tasks and focused on the parietally distributed P300 component in clinical or inmate populations (e.g., Harmon-Jones et al., 1997; Stanford et al., 2003). However, some studies have examined healthy adult populations (e.g., Gerstle et al., 1998; Mathias and Stanford, 1999).

The results from these studies largely suggest that higher self-reported impulsive aggression and hostility were associated with reduced parietal and/or central P300 amplitude (Harmon-Jones et al., 1997; Gerstle et al., 1998; Mathias and Stanford, 1999). The amplitude of the P300 is thought to reflect information processing capacity including stimulus evaluation, attention allocation, and context updating (e.g., Donchin and Coles, 1988; Coles et al., 2000). These results suggest that aggressive individuals may have impairments in these cognitive abilities.

Stewart et al. (2010) extended these results using a sample of 48 men and 54 women. The authors showed that higher anger-out scores were associated with increased P300, N200 (indicating increased response inhibition and/or conflict monitoring), and N400 (indicating increased elaborative stimulus processing) amplitude to negative words. The N200 and N400 are fronto-centrally distributed components of the ERP. These findings suggest that aggressive individuals may exert more effort to override attention to negative information (Stewart et al., 2010). Further, higher anger-in predicted decreased N400 amplitude to negative words, suggesting that these individuals need fewer attentional resources to suppress negative stimuli (Stewart et al., 2010).

##### State Anger/Aggression and ERPs

Other studies have investigated how inducing anger or aggression affects ERPs (e.g., Krämer et al., 2008; Gable and Poole, 2014). Only one study investigated gender differences (Krämer et al., 2008). In this study, 25 men and 24 women were provoked within the TAP. When participants were deciding on the volume of a shock they would deliver to an opponent, participants high in trait aggression showed enhanced frontal negativity (i.e., N200) when the opponent delivered a high noise blast compared to a low noise blast (Krämer et al., 2008). This effect was greater in participants high in trait aggression who behaved less aggressively in the task. These results suggest that participants higher in trait aggression were more prone to detect conflict and attempted to exert inhibitory control. Men and women did not differ on neurophysiological responses. Another study suggests that women high in trait hostility showed a pattern of EEG data that is compatible with heightened emotional responding to emotional faces but also heightened inhibitory control (Knyazev et al., 2009). Men high in hostility did not show the inhibitory control effect, which is consistent with gender differences in aggressive behavior.

#### Brain Stimulation

Frontal cortical asymmetry can be induced with transcranial magnetic stimulation (TMS) and transcranial direct current stimulation (tDCS; for reviews see Angus et al., 2016; Kelley et al., 2017). Slow repetitive TMS (rTMS) can inhibit cortical excitation. Using a small group of 10 healthy women, one study found that inhibiting the right PFC using rTMS caused selective attention toward angry faces, whereas inhibiting the left PFC caused selective attention away from angry faces (d’Alfonso et al., 2000). Similar patterns of frontal activation results have been observed in predominantly female samples (van Honk and Schutter, 2006; Hofman and Schutter, 2009). These results should be interpreted cautiously, however, as another study that used continuous theta-burst magnetic stimulation (a form of TMS) found contrary results in a predominantly male sample. Results showed that inhibition of the left dorsolateral prefrontal cortex (DLPFC) increased aggression compared to inhibition of the right DLPFC (Perach-Barzilay et al., 2013). These results might reflect methodological factors rather than gender effects. For example, the latter study specifically targeted the DLPFC rather than the broader PFC.

Applications of tDCS to the PFC have also found mixed results using healthy samples. One study found that increasing relative left frontal activation increased behavioral aggression after provocation when participants were angry (40 men, 40 women; Hortensius et al., 2012). Riva et al. (2015) found consistent results with Hortensius et al. (2012) in a predominantly female sample (*n* = 63/80). Specifically, increasing relative right activation in the ventrolateral prefrontal cortex (VLPFC) decreased aggression after social exclusion compared to sham stimulation (Riva et al., 2015). Another study examined 13 men and 19 women and induced right hemispheric dominance via tDCS to the DLPFC (Dambacher et al., 2015a). The stimulation decreased unprovoked aggression, but only in men. The tDCS did not reduce provoked aggression in men or women. A similar study did not find any gender differences when examining the effect of bilateral tDCS on response inhibition or aggression (39 men, 25 women; Dambacher et al., 2015b). Additional research would help to clarify how increasing relative left or right frontal activation impacts anger and aggression and the role of gender in these relationships.

#### Bodily Manipulations

Hand contractions and body positioning can induce asymmetric frontal activity. Contracting the left hand increases relative right frontal activity while contracting the right hand increases relative left frontal activity (Harmon-Jones, 2006). In a study of all women (*N* = 43), following an insult, women who contracted their right hand assigned louder and longer noise blasts to the provocateur than women who squeezed their left hand (Peterson et al., 2008). Relative left frontal activity positively correlated with behavioral aggression for women who squeezed their right hand.

Another study using both men and women found that right hand contractions caused not only greater relative left frontal activity, but also greater self-reported anger in response to ostracism (Peterson et al., 2011). The authors reported that these effects did not differ between men (*n* = 9) and women (*n* = 17). Further, in an equal sample of men and women (*n*s = 23), relative to sitting in an upright position and/or leaning forward, being in a supine position reduced relative left frontal activation in response to an anger-evoking event (Harmon-Jones and Peterson, 2009).

#### Summary

Overall, these EEG/ERP and frontal asymmetry manipulation studies provide insight into the neural activation associated with anger and aggression. Although studies often included both men and women, only a select few investigated potential gender differences in these effects. Of those that did, most revealed no differences between men and women and were underpowered. More research is warranted to directly test the nature of gender effects in frontal asymmetry, ERPs, brain stimulation, and bodily manipulations. There is no evidence of robust gender differences in EEG and most studies did not report testing for gender differences.

#### Neuroimaging Studies

Several fMRI studies examined neural responses during aggression paradigms in men and women and less commonly, in women only. These studies primarily used the TAP. The methods, analyses, and results differ somewhat from study to study. However, the general consensus is that behaving aggressively activates brain regions associated with negative affect, arousal, cognitive-behavioral control, mentalizing and reward. Specifically, these studies observed activation in the DLPFC, VLPFC, medial prefrontal cortex (MPFC), anterior cingulate (ACC), amygdala, putamen, caudate, thalamus, insula, ventral striatum and hippocampus (Krämer et al., 2007; Lotze et al., 2007; Chester and DeWall, 2016; Emmerling et al., 2016).

None of these studies tested hypotheses about gender differences, but several studies did include both men and women. For instance, one of the first fMRI studies to examine neural activity during the TAP included 11 men and 11 women (Krämer et al., 2007). Another study of 11 women and 9 men found that provocation during the PSAP elicited activation in the ACC, dorsal striatum, insula and PFC (Skibsted et al., 2017). This provocation-related activation correlated with aggressive behavior in the paradigm (i.e., stealing points).

Another study of 30 healthy undergraduate women measured startle eyeblink responses to neutral (e.g., household items) vs. threatening images (e.g., a gun pointed at the participant; Beyer et al., 2014). No men were included in the study. Women with relatively greater startle responses to threatening over neutral images were considered fearful and reactive to threat. Results showed that women with relatively greater startle responses showed lower activation in the brain’s mentalizing network, which includes the dorsomedial prefrontal cortex (DMPFC). Corroborating evidence suggests that the DMPFC is positively correlated with aggressive behavior and angry rumination, likely stemming from hostile mentalizing (Lotze et al., 2007; Denson et al., 2009). The authors concluded that women with greater threat reactivity engaged in less mentalizing than women low in threat reactivity. These findings are consistent with meta-analytic reviews showing women’s greater feelings of danger and fear when provoked (Eagly and Steffen, 1986; Ito et al., 1996).

Additional fMRI studies examined men and women with borderline personality disorder, which is characterized by reactive aggression (Lieb et al., 2004). A structural MRI study examined the relationships between right and left amygdala volumes with trait aggression in men and women with borderline personality disorder and healthy controls (Mancke et al., 2016). Borderline women reported greater trait aggression than healthy women, but there was no relationship between amygdala volumes and trait aggression in either group of women. By contrast, men showed a positive correlation between right amygdala volume and trait aggression, but only among those diagnosed with borderline personality disorder. Thus, amygdala volume may not be an important factor in aggression among women with borderline personality disorder.

In an fMRI study on borderline personality, women, men and healthy controls engaged in a script-driven imagery task that consisted of two phases (Herpertz et al., 2017). In the anger phase, participants listened to recorded scripts describing harsh interpersonal rejection. Next, in the aggression phase, participants listened to a script describing aggressive behavior. Participants were asked to fully immerse themselves in the scripts. As in the previous study, women with borderline personality disorder reported greater trait aggression and trait anger than healthy women. During both the anger and aggression portions of the task, there were no differences in any of the regions of interest between borderline and healthy women. However, during the aggression phase, women with borderline personality disorder showed positive connectivity with the amygdala and middle cingulate cortex. Men showed the opposite effect; negative connectivity between the amygdala and middle cingulate cortex. Trait anger, but not trait aggressiveness, further strengthened this connectivity in women and weakened it in men. Thus, when imagining an aggressive act, dispositionally aggressive women showed greater amygdala-cingulate connectivity than their male counterparts.

Using another type of social provocation, 15 women and 15 men played a ball tossing game (i.e., Cyberball), ostensibly with two other fictitious same sex participants (Chester and DeWall, 2016). Participants are eventually ignored and left out of the game. This form of ostracism increases anger, aggression and activation in the dorsal anterior cingulate cortex (dACC). In this study, participants completed a measure of trait narcissism followed by playing Cyberball in the scanner. Afterwards outside of the scanner, they were allowed to retaliate via the TAP against one of the two fictitious players. Results showed that the most aggressive participants reported high narcissism and also showed a large increase in the dACC. No gender effects were reported, but they did note that controlling for gender strengthened the effect size of the interaction.

Using the same Cyberball social exclusion method, 20 women and 14 men were either included in the game or excluded (Beyer et al., 2014). Afterwards, participants completed the TAP followed by viewing neutral and emotional scenes. Excluded participants showed heightened activation to emotional social scenes in the brain’s mentalizing network, including the DMPFC. In excluded participants, activation in the precentral gyrus in response to viewing emotional scenes mediated the effect of exclusion on aggressive behavior.

##### Neuroimaging Studies of Substance Use and Aggression

Researchers are beginning to use fMRI to investigate brain mechanisms responsible for aggression related to alcohol and illicit drugs. Because methamphetamine dependence is associated with increased aggression, Payer et al. (2011) investigated aggression-related neural activity in this population (16 women, 23 men) and healthy controls (18 women, 19 men). Participants completed an affect matching and an affect labeling task. During the affect matching task, participants selected an emotional facial expression that matched a target image. During the labeling task, participants verbally labeled the emotional facial expression. During affect matching, methamphetamine dependent participants showed less activation than controls in the ventral inferior frontal gyrus. During labeling, both dependent and control participants showed increases in the dorsal inferior frontal gyrus and decreases in amygdala activity. Larger amygdala decreases were correlated with lower aggression in the TAP outside of the scanner. Although the authors noted significant gender differences in gray matter volume in the inferior frontal gyrus and amygdala, they did not describe the nature of those differences.

Two fMRI studies investigated the neural correlates of alcohol-related aggression in men and women. In one study, 13 formerly alcohol-dependent participants and 13 controls completed the PSAP in the scanner (Kose et al., 2015). When provoked, control participants showed greater activation in the PFC, thalamus and hippocampus than the formerly dependent group. Independent of group, participants showed negative correlations between the orbitofrontal cortex (OFC), PFC, caudate and thalamus and aggressive behavior. However, these results should be interpreted cautiously as there were only three women in the formerly alcohol dependent group and six in control group.

Another study examined the effects of acute alcohol intoxication on aggression and neural responses (Gan et al., 2015). In that study, 24 healthy young men and 11 women completed the TAP in the scanner once while intoxicated and once after consuming a placebo. Alcohol decreased BOLD responses in the right PFC (i.e., middle frontal and inferior frontal gyri), hippocampus, thalamus, caudate and putamen. Moreover, activity in the amygdala and ventral striatum was not affected by alcohol but was positively correlated with aggression against the provoking opponent. Gender did not influence any of the results, but the authors noted that additional research is needed due to the small sample size. Another study of 12 women and 10 men did not examine aggression but did find that alcohol reduced frontal connectivity in women but not men (Hoppenbrouwers et al., 2010). This frontal dysregulation may be one possible pathway to aggression in women.

#### Summary

Neural mechanisms underlying aggression remain poorly understood in women. As most studies did not investigate gender differences and were underpowered, there is not enough evidence of different neural pathways for men and women. The small sample sizes, few women, reliance on the TAP or PSAP, and diverse results preclude firm conclusions at this point. Additional fMRI studies with large samples of men and women and diverse aggression tasks are needed.

#### Hormones

In the realm of aggressive behavior, testosterone, cortisol, estradiol, progesterone and oxytocin have been studied extensively in non-human animals, but less so in humans. In this section, we review the evidence on the relationships between these hormones and aggression in women.

##### Testosterone and Cortisol

In mammalian species, males generally have higher testosterone levels and are more aggressive than females. Similarly, because men are more violent than women globally and men possess much higher testosterone concentrations than women, researchers suspected that testosterone is a strong cause of aggression in men. However, much less research has investigated this possibility in women. One study of 87 women inmates in a maximum-security prison found that testosterone levels correlated with aggressive dominance in prison (Dabbs and Hargrove, 1997). This relationship was reduced among older women, presumably due to lower levels of testosterone. Similarly, a study of a women’s rugby team found that the pre-game rise in testosterone was positively correlated with aggressiveness during the game (Bateup et al., 2002). Another correlational study measured testosterone in 155 men and 151 undergraduate women (Harris et al., 1996). Men reported greater aggression than women and had five times more testosterone than the women. Despite these mean differences, the authors found positive correlations between testosterone and self-reported aggression in both women and men. Thus, although aggression and testosterone may be lower in women than men, many studies observed the same positive relationships between testosterone and aggression in women as they do in men (e.g., Prasad et al., 2017; Probst et al., 2018). A study of 12 women in a double-blind placebo-controlled testosterone administration study suggests that testosterone may increase aggression because it reduces sensitivity to punishment and increases reward sensitivity (van Honk et al., 2004).

A meta-analysis revealed that the correlations between testosterone and aggression were small, but significant in both men (*r* = 0.08) and women (*r* = 0.13; Archer et al., 2005). Thus, the relationship between testosterone and aggression is not particularly strong in humans. Indeed, a review of the literature suggested that testosterone should be considered as promoting dominance seeking behavior, rather than solely aggression (Eisenegger et al., 2011).

In order to explain these weak correlations between testosterone and aggression, researchers examined cortisol as a moderator of this relationship. The dual hormone hypothesis suggests that low cortisol facilitates the potentiating effect of testosterone on aggressive and dominant behavior, whereas high cortisol blocks this effect (Mehta and Prasad, 2015; for a similar notion using the ratio of testosterone to cortisol, see Terburg et al., 2009). This pattern of data has been observed in forensic samples of men and boys (Dabbs et al., 1991; Popma et al., 2007), but evidence is mixed in women. For instance, one study of 53 healthy undergraduate women found the opposite pattern; women with high concentrations of both salivary testosterone and cortisol showed the most aggression in the TAP (Denson et al., 2013). Other studies failed to find support for the dual hormone hypothesis in women (Cote et al., 2013; Geniole et al., 2013; Welker et al., 2014; Buades-Rotger et al., 2016). However, a recent study of 326 adolescent girls and 134 boys found that testosterone derived from hair samples correlated with self-reported aggression at low levels of cortisol in both boys and girls (Grotzinger et al., 2018). Estimates derived from hair samples may reflect stable trait-like individual differences in cortisol and testosterone more so than values derived from saliva. Thus, these data suggest that interactions between testosterone and cortisol may influence aggression in women. However, more research is needed with large samples and behavioral measures of aggression.

The dual hormone serotonergic hypothesis goes one step further by positing that the interactive relationship between testosterone and cortisol on aggression is further moderated by serotonin availability (Montoya et al., 2012). Specifically, high testosterone, low cortisol, and low serotonin are thought to increase risk for aggression. One study did examine the interactive effects of testosterone and serotonin on trait aggression in 24 women and 24 men (Kuepper et al., 2010). Participants provided testosterone samples over 3 days and subsequently received S-citalopram. The dependent variable was trait aggression. S-citalopram influences serotonin and cortisol. A large vs. small cortisol response to the drug is thought to indicate high vs. low 5-HT availability, respectively. Only men showed the expected high testosterone-low serotonin interaction on trait aggression. Unexpectedly, they also found a low testosterone-high serotonin interaction. Thus, more research is needed to verify the robustness of these results and their applicability to women.

Although most research on hormones and aggression is correlational, some researchers have conducted placebo-controlled experiments. In one such study 24 women and 24 men were administered cortisol or a placebo and subsequently exposed to strong or weak provocation within the TAP (Böhnke et al., 2010). Cortisol increased aggression in women but not men, but only during the most provocative trials of the TAP. Results should be interpreted cautiously due to small cell sizes.

Other research investigated relationships between hormones and neural activity. For instance, Mehta and Beer (2010) found that in a sample of 17 men and 15 women, endogenous testosterone positively correlated with aggression during the Ultimatum Game and negatively with bilateral medial OFC activation. Medial OFC activity statistically mediated the relationship between testosterone and aggression. There were no differences between men and women. However, another fMRI study found a negative relationship between testosterone and aggression in an all-female sample of 39 undergraduates (Buades-Rotger et al., 2016). In that study, participants were exposed to an opponent’s angry face or neutral face followed by provoking noise blasts. Testosterone was negatively correlated with amygdala reactivity to the trials with an angry face. Thus, much more research is needed on hormones and neural responses before firm conclusions can be made about these mechanisms in women.

##### Estradiol and Progesterone

In women, the two ovarian hormones estradiol and progesterone reliably fluctuate during the menstrual cycle. Peak fertility is characterized by high levels of estradiol and low levels of progesterone. Gladue (1991) examined the relationships between estradiol, testosterone, and trait aggression in a matched sample of heterosexual and same-sex attracted men and women. Regardless of sexual orientation, both testosterone and estradiol positively correlated with trait aggression in men; for women, these correlations were negative. Another study of 49 undergraduate women found no relationship between testosterone and trait aggression but replicated the negative relationship between estradiol and trait aggression (Stanton and Schultheiss, 2007).

In another study, 34 undergraduate women kept diaries of competition-related conflict and how they dealt with it (Cashdan, 2003). Women relatively high in testosterone were more likely to resolve the conflict with verbal aggression. Estradiol was unrelated to aggression. Similarly, a study of 33 bulimic women and 23 healthy controls in the early follicular phase of the menstrual cycle reported a positive association between testosterone and trait aggression, but only in the bulimic group (Cotrufo et al., 2000). No correlations were found between estradiol, prolactin and cortisol in either group.

Collectively, these data suggest that endogenous estradiol may be either unrelated or negatively related to aggression in women. However, estradiol may be involved in dominance, assertiveness, and risk-taking in women rather than aggression. Estradiol is positively correlated with implicit power motivation (for a replication see, Stanton and Edelstein, 2009). Similarly, we found that high estradiol and low progesterone was associated with heightened assertiveness in women (Blake et al., 2017). High levels of free estradiol were positively correlated with both aggressive and non-aggressive risk-taking (Vermeersch et al., 2008).

Relatively few studies tested the hypothesis that progesterone would be related to aggression. Ritter (2003) measured trait aggression in 29 healthy undergraduate women during menses and again during the midluteal phase. Progesterone and estrogen are higher during the midluteal phase than during menses. Women reported less trait physical and verbal aggression during the midluteal phase than during menses. However, this study did not directly measure hormones so it is unclear whether the menstrual cycle effect on trait aggression was due to estradiol, progesterone, or both.

Another study measured estradiol, progesterone and testosterone across the menstrual cycle in 15 healthy women (Brambilla et al., 2010). They found positive correlations between estradiol and verbal aggression during the follicular phase, when progesterone and estradiol are low. Testosterone was uncorrelated with hostility and aggression. They also found a negative correlation between progesterone and two components of trait hostility (i.e., suspiciousness and resentment) in the luteal (premenstrual) phase. This finding was conceptually replicated in a larger sample of 122 women (Ziomkiewicz et al., 2012). They found that higher levels of progesterone during the luteal phase were associated with lower self-reports of aggression and irritability. Thus, greater progesterone may reduce hostility and aggression during the luteal phase, whereas low levels of progesterone may increase risk for aggression.

Simultaneously low levels of progesterone and estradiol may increase self-directed aggression. Indeed, one study examined estradiol and progesterone in 281 fertile women within 24 h after attempting suicide (Baca-Garcia et al., 2010). Suicide attempts were more likely during periods of low estradiol and progesterone. Thus, progesterone may be protective against both other-directed and self-directed aggression. One possibility is that progesterone may be associated with improved emotion regulation capacity. In an attempt to determine how high levels of progesterone may aid emotion regulation, 18 healthy women completed an emotion matching task during fMRI with angry and fearful faces (van Wingen et al., 2008). Relative to placebo, a single progesterone administration increased amygdala activity and connectivity between the amygdala and dACC. This latter finding raises the possibility of progesterone assisting emotion regulation via connectivity between the dACC and amgydala (for a review of neuroimaging findings, see Toffoletto et al., 2014).

##### Oxytocin

Although sometimes referred to as the “love hormone” or “bonding hormone”, the nonapeptide oxytocin may also increase aggressive behavior. Most studies examining oxytocin have either intranasally administered the hormone or a placebo. Less frequently, researchers obtain endogenous levels via lumbar puncture. One study found that oxytocin levels measured in the cerebrospinal fluid were negatively correlated with trait aggression in women (*n* = 13; Lee et al., 2009). Similarly, Campbell and Hausmann (2013) found that oxytocin relative to placebo lowered aggression on the PSAP, but only among women who were feeling anxious.

Breastfeeding women typically have high levels of oxytocin. One laboratory study using the TAP found that breastfeeding women were more aggressive than formula feeding women and nulliparous women (Hahn-Holbrook et al., 2011). The greater aggression in breastfeeding women relative to the other women was due to lowered stress responses to provocation among the breastfeeding women. Thus, oxytocin may facilitate aggression by lowering perceptions of danger that normally inhibit many women from retaliating (Bettencourt and Miller, 1996). Thus, oxytocin may both increase and decrease aggression via reduced anxiety.

Consistent with this possibility, an fMRI study of 38 women with borderline personality disorder and 41 healthy women were given oxytocin or a placebo (Bertsch et al., 2013). They then classified emotional facial expressions while in the scanner. Relative to the borderline women in the placebo group, borderline women given oxytocin showed reduced threat sensitivity to angry faces and lower amygdala activation. These findings are consistent with the studies showing anxiolytic effects of oxytocin in women and the possibility that oxytocin influences aggression via reduced fear (Campbell, 2008).

In order to make sense of conflicting results of oxytocin on social behavior, Shamay-Tsoory and Abu-Akel (2016) proposed the social salience hypothesis. The idea is that oxytocin enhances the perception of social stimuli; thus, enhancing responses to both positive and negative (e.g., provocation) social stimuli. In this way, provoking individuals should be perceived as more hostile following oxytocin administration. A recent study found support for the social salience hypothesis in a laboratory experiment of 28 men and 20 women (Ne’eman et al., 2016). Using a modified version of the PSAP, participants could behave selfishly, cooperatively, or aggressively. Relative to placebo, oxytocin selectively increased aggressive responses. The authors found no gender differences.

Consistent with the social salience hypothesis, other work suggests that oxytocin may increase IPV. In a placebo-controlled experiment, 46 women and 47 men received oxytocin or placebo, after which they completed a physical pain task and received negative social feedback on a speech (DeWall et al., 2014). Next, they reported on how likely they would be to commit physical IPV against their current partner (or former partner for the single participants). Results showed that oxytocin increased IPV inclinations, but only for those high in trait aggression. Women reported greater IPV inclinations than men, but gender did not interact with the oxytocin manipulation. The authors suggested that people high in trait aggression may engage in more IPV as a controlling tactic when experiencing negative affect. However, there is another plausible alternative explanation that is consistent with the social salience hypothesis. Oxytocin may have enhanced the subjective impact of the pain and negative feedback. Among people high in trait aggression, who tend to have a hostile world view, this greater oxytocin-induced impact may have facilitated greater inclinations towards IPV (Buss and Perry, 1992).

#### Summary

This brief review of five hormonal mechanisms underlying aggression in women suggests few clear findings. As with men, the positive relationship between testosterone and aggression in women is small. The dual hormone hypothesis has had some success in predicting aggression in men, but less so in women. The data on estradiol and progesterone are suggestive of the possibility that high levels of these hormones reduce aggression and self-directed harm in women. However, much more work is needed. The literature on oxytocin suggests that the hormone can decrease and increase aggression in women. Increases in aggression are likely due to a combination of the hormone’s anxiolytic effects as well as enhanced reactivity to provocation. The social salience hypothesis provides a promising framework from which to test specific predictions about conditions under which oxytocin enhances or inhibits aggression in women.

## Discussion

In this review, we examined the numerous behavioral expressions of aggression that women engage in along with the early developmental, neural, and hormonal correlates. The factors are summarized in Figure 1. Our review highlights that relative to men’s aggression, we know little of the underpinnings of women’s aggression. Most studies on brain and hormonal mechanisms of aggression included only men, did not examine gender differences, or did so in a *post hoc* manner, and/or relied on small samples. Thus, there is little opportunity to make robust conclusions about how the processes reviewed here influence aggression in women. By contrast, the behavioral data are clear in that women tend to engage in predominantly indirect aggression, IPV with equal frequency but lesser severity than men, and rarely sexual aggression. Thus, our review is in accord with Richardson (2005), who noted that women are quite capable of aggression. Nonetheless, the limitations of the extant data provide opportunities for future research testing novel hypotheses. We urge more theoretical development to derive *a priori* gender-specific predictions about the mechanisms underlying aggressive behavior in women.

### Future Directions

There are a number of unknown aspects about the causes and nature of women’s aggression. For instance, little is known about aggression in same-sex attracted women. Relative to men, the perpetration of sexual aggression in women remains poorly understood as well. Sexual aggression committed by women is a relatively low frequency behavior and victims are unlikely to report its occurrence. These issues make it a difficult phenomenon to study. Nonetheless, both men and women victims of sexual violence show the same negative psychological outcomes, making all forms of sexual violence worthy of further study. Laboratory sexual aggression paradigms developed for women would be informative (see Davis et al., 2014).

Our review of neural correlates of aggression also showed no convincing evidence of divergent pathways for men and women. Most of the EEG/ERP, brain stimulation, and fMRI studies that included men and women did not report testing for gender differences or did not find any. The role of hormones in determining women’s aggression was also largely unclear, but worthy of future study as theoretical development in this area is becoming increasingly sophisticated (Mehta and Prasad, 2015; Shamay-Tsoory and Abu-Akel, 2016). Since fear plays a significant role in women’s reaction to provocation and subsequent aggression (Eagly and Steffen, 1986; Bettencourt and Miller, 1996), brain regions involved in fear processing and arousal (e.g., amygdala, hypothalamus) seem like promising regions of interest.

One limitation of the laboratory and brain research on women’s aggression is the reliance on the TAP and PSAP as the primary measures of aggression. Although well-validated, both involve direct retaliation toward the provocateur. Women tend to engage in indirect aggression to a greater extent than direct aggression. Thus, it is unclear to what extent the laboratory work represents realistic behavior in women. Development of indirect aggression paradigms for the laboratory would facilitate greater understanding as would field experimentation.

We have also left out a discussion of genetic influences. Aggression is highly heritable, and in the past several years, a number of candidate genes such as *MAOA* and *5-HTTLPR* have been identified as conferring risk for aggression, impulsivity, and emotion regulation deficits (Ficks and Waldman, 2014). Similarly, the field of epigenetics has much to offer, especially if we are to understand women’s aggression across the lifespan (Waltes et al., 2016). Optogenetic technology in animal models also holds promise. For instance, optogenetic stimulation of neurons in the hypothalamus caused male mice to attack females, males, and inanimate objects (Lin et al., 2011). Using optogenetics holds promise for understanding some of the brain processes that may heighten female aggression.

Although it was outside of the scope of this review, all the mechanisms we discussed here are mediated via neurobiological processes that we did not discuss. For instance, serotonin has been robustly implicated in aggression and is affected by prenatal smoking and maternal malnutrition (Liu, 2011). There are no doubt many mediating processes at various levels of specificity that remain to be explored.

## Conclusion

Aggression is a complex social behavior that has been extensively studied in men. Comparatively, women’s aggression has been neglected. We suggest that there is a need for more theory-driven research in the investigation of aggression in women. Such work could contribute to the development of more effective evidence-based treatments that target gender-specific risks for aggression.

## Author Contributions

TFD drafted the sections on laboratory aggression, sexual aggression, prenatal influences, neuroimaging and hormones. He also drafted the general discussion. SMO drafted the intimate partner violence section. JRB drafted the EEG, brain stimulation and bodily manipulations sections. KRB wrote portions that appear throughout the manuscript. All authors provided critical revisions and contributed to theoretical development.

## Conflict of Interest Statement

The authors declare that the research was conducted in the absence of any commercial or financial relationships that could be construed as a potential conflict of interest.

## Footnotes

^1^The Eagly and Steffen (1986) and Bettencourt and Miller (1996) meta-analyses also included field studies that contained a behavioral measure of aggression.
